# Application of In Vitro Metabolism Activation in High-Throughput Screening

**DOI:** 10.3390/ijms21218182

**Published:** 2020-10-31

**Authors:** Masato Ooka, Caitlin Lynch, Menghang Xia

**Affiliations:** National Center for Advancing Translational Sciences, Division for Pre-Clinical Innovation, National Institutes of Health, Bethesda, MD 20892, USA; masato.ooka@nih.gov (M.O.); caitlin.lynch@nih.gov (C.L.)

**Keywords:** CYPs, in vitro metabolism, HTS, hepatocytes, HepaRG, 3D cell culture, organoids, organ-on-a-chip

## Abstract

In vitro methods which incorporate metabolic capability into the assays allow us to assess the activity of metabolites from their parent compounds. These methods can be applied into high-throughput screening (HTS) platforms, thereby increasing the speed to identify compounds that become active via the metabolism process. HTS was originally used in the pharmaceutical industry and now is also used in academic settings to evaluate biological activity and/or toxicity of chemicals. Although most chemicals are metabolized in our body, many HTS assays lack the capability to determine compound activity via metabolism. To overcome this problem, several in vitro metabolic methods have been applied to an HTS format. In this review, we describe in vitro metabolism methods and their application in HTS assays, as well as discuss the future perspectives of HTS with metabolic activity. Each in vitro metabolism method has advantages and disadvantages. For instance, the S9 mix has a full set of liver metabolic enzymes, but it displays high cytotoxicity in cell-based assays. In vitro metabolism requires liver fractions or the use of other metabolically capable systems, including primary hepatocytes or recombinant enzymes. Several newly developed in vitro metabolic methods, including HepaRG cells, three-dimensional (3D) cell models, and organ-on-a-chip technology, will also be discussed. These newly developed in vitro metabolism approaches offer significant progress in dissecting biological processes, developing drugs, and making toxicology studies quicker and more efficient.

## 1. Introduction

Metabolism is an essential biological process that transforms food into energy, converts food to basic building blocks (i.e., amino acids), and eliminates toxic compounds from the body. As metabolism proceeds, it will activate or inactivate a parent compound into its metabolites with more (activation) or less (deactivation) activity. With rapidly increasing numbers of chemical compounds being introduced into our environment, there is a great need for more efficient and trustworthy screening methods to evaluate the safety of each compound. To investigate how metabolites act inside our bodies, screening systems require in vitro metabolism methods. However, there are several challenges when incorporating metabolism into high-throughput screening (HTS) assays. For instance, most immortalized and engineered stable cell lines have little to no metabolic capability [1]. Some primary cells, such as hepatocytes, have metabolic activity; however, these cell lines usually have limited tissue/organ-specific metabolic enzymes [2,3], providing an organ-limited prediction value. Moreover, the metabolic activity encompassed in these cell lines might not be enough to detect the weaker activating chemicals. Recently, efforts have been put forth to improving assays with metabolic capability, such as stimulating metabolism in vitro by the addition of human and rat liver microsomes [4] or using cell lines with enhanced metabolism enzymes generated by genetic modification [5]. In this review, we describe several in vitro metabolism methods and their ability to be used in an HTS platform [6].

### 1.1. Phase I and Phase II Metabolism

Most chemicals are broken down inside cells by enzymes in phase I and/or II of drug metabolism. Phase I enzymes take part in oxidation, reduction, and hydrolysis [7,8]. In short, phase I reactions convert the parent compound into a more hydrophilic metabolite by adding -OH, -SH, or -NH_2_ groups to the end of a compound [8]. This modification leads to activation, in the case of a prodrug, or inactivation of a parent drug [8]. Cytochrome P450 (CYP450) enzymes, belonging to a superfamily of enzymes containing heme as a co-factor and which function as monooxygenases, play an essential role in phase I of drug metabolism. The CYP family consists of 57 genes in humans [9] and takes part in 75% of total small molecule drug metabolism [10]. CYPs are the majority of phase I enzymes and are vital in metabolism for most xenobiotics. They also take part in several endogenous pathways, such as steroid hormone biosynthesis [11,12]. Since the liver is primarily responsible for metabolism, hepatocytes express an abundance of CYP enzymes and demonstrate the highest capacity for phase I xenobiotic biotransformation [13]. The amount and activity of these CYPs are mainly regulated by a class of transcription factors called nuclear receptors (NRs) [14].

Phase II enzymes conjugate the drug with an endogenous charged compound such as sulfate, glucuronide, glutathione, or an amino acid [8]. The conjugated metabolite will then have a larger size and increased water solubility. Phase II reactions are catalyzed by transferases that require co-factors [15]. For example, glucuronosyltransferase (UGT) transfers the co-factor, UDP-glucuronic acid, to the drug to create a conjugated compound. Some common medications metabolized by UGT include acetaminophen, morphine, lamotrigine, oxazepam, and temazepam [16].

### 1.2. Drug Metabolism and Drug-Drug Interactions

**Figure 1:**
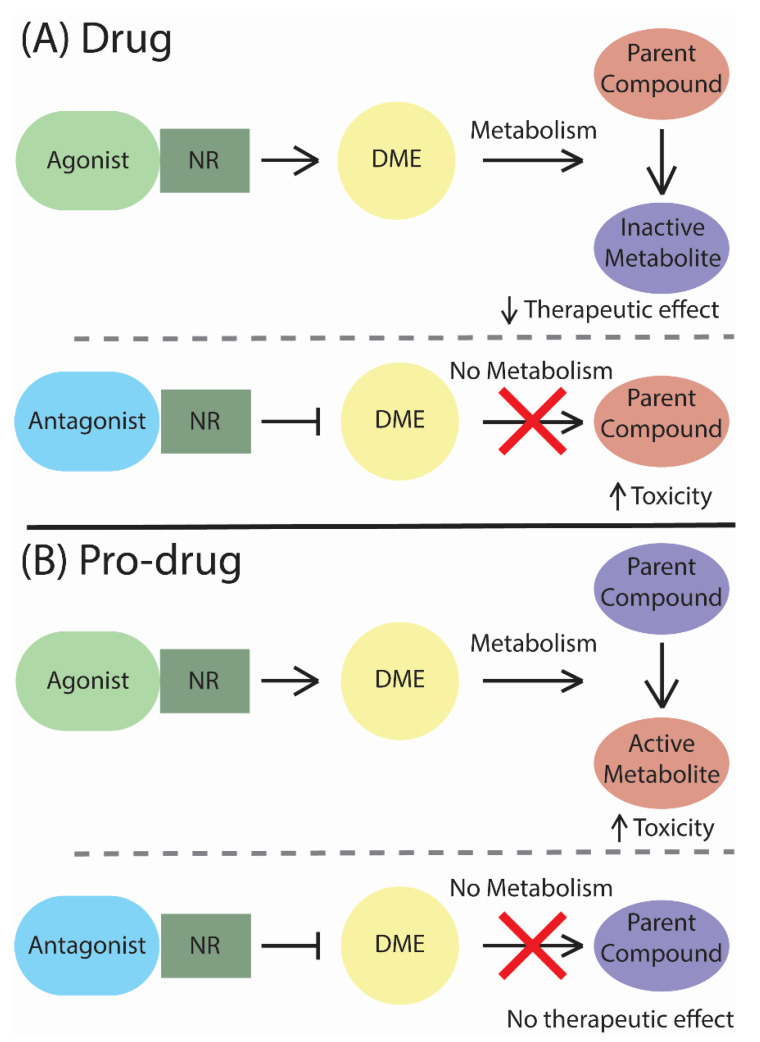
The metabolic scheme of drugs and prodrugs. (**A**) A nuclear receptor (NR) agonist activates the expression of drug-metabolizing enzymes (DMEs), leading to a decrease in activity of the drug, while an NR antagonist deactivates the expression of DMEs, keeping the drug active. (**B**) An NR agonist activates the expression of DMEs, leading to prodrugs becoming activated. An NR antagonist deactivates the expression of DMEs, keeping a prodrug inactive.

Drug-drug interactions (DDIs) occur when two or more drugs are used simultaneously, resulting in a change in the efficacy of either drug (Figure 1) [17]. One of the critical aspects of NRs, or transcription factors in general, is the ability to mediate DDIs by transcribing drug-metabolizing enzymes (DMEs) and transporters. Some drugs may activate or deactivate NRs, leading to a change in the activity of enzymes and transporters in the body. The most highly expressed CYP in the liver is CYP3A4, followed by CYP2C9, CYP2D6, and CYP2B6 [18,19]. CYP3A4 expression is regulated, in part, by the pregnane X receptor (PXR), which is part of an NR superfamily. The activation of PXR will induce CYP3A4 transcription, enhancing the metabolizing potential of this DME [20,21]. Another NR which can cause multiple DDIs is the constitutive androstane receptor (CAR), due to its transcriptional regulation of multiple DMEs, such as CYP2B6 and, to a lesser extent, CYP3A4 [19].

The most common types of DDIs are the inhibition or induction of DMEs [22]. These interactions can increase or decrease the efficiency of a drug when co-treatment with other drugs is employed. As mentioned before, drugs can affect NR activity, causing an unexpected change in drug metabolism. One drug may increase another drug’s metabolism by increasing the DMEs via NR modulation. As a result, the drug metabolism rate increases, often causing a decreased therapeutic effect of the drug [23]. However, in the case of prodrugs, the decrease in phase I metabolism will decrease the activity of the drug (Figure 1) [24,25]. When a drug decreases the activity of DMEs, a co-treated drug can become toxic to the body due to the inhibition of metabolism. NR modulation from drugs can also inhibit or induce transporters, leading to a decreased or increased amount, respectively, of drugs being carried.

### 1.3. Role of Transcription Factors in CYP450 Regulation

In the mid-1980s, the NR field entered the scientific world and identified this new superfamily as having at least 48 members in humans [26]. All NRs are known transcription factors and were found to have five main parts to their structure: an *N*-terminal domain, a DNA-binding domain, a hinge region, a ligand-binding domain, and a C-terminal domain. The differences in DNA-binding domains and ligand-binding domains generate the vast array of roles with which NRs react in the body. A diverse array of ligands, such as endogenous hormones or exogenous chemicals, can directly or indirectly activate or deactivate these important proteins [27]. Once modulated, each NR has a different role and mechanism in the human body causing a change in the transcription of different proteins and can therefore be a potential drug target.

The main receptors, when discussing metabolism, drug or otherwise, are the orphan nuclear receptors. They are so-called due to their initial discovery being done without the identity of an endogenous ligand. Some of these orphan NRs regulate key genes involved in lipid, bile acid, and cholesterol sensing, such as the liver X receptor and farnesoid X receptor [28]. However, the major xeno-sensing NR is PXR. This important NR regulates many different CYPs, with the predominant one being CYP3A4, the enzyme involved in an estimated 50% of drug metabolism [29]. PXR is also involved in the regulation of other DMEs and transporters, such as CYP2Bs, CYP2Cs, UGT1A1, multi drug response 1 (MDR1), and multidrug resistance-associated protein 3 (MRP3), as well as other NRs, such as CAR [30]. This large variation in target genes makes PXR one of the most important NRs to study when determining the activity of drug metabolism.

Another critical NR in the regulation of DMEs, and therefore DDIs, is CAR, PXR’s sister receptor. Inside the liver, CAR can be found in the cytoplasm of the cell until activation shuttles the NR into the nucleus. However, this unique orphan NR has constitutive activity in immortalized cells, meaning CAR is found inside the nucleus and is already activated without a ligand present. This makes screening for modulators a difficult task, and requires the use of liver cells or a selective antagonist to reverse the constitutive activity [31]. Interestingly, CAR also regulates CYP3A4 to an extent, but majorly regulates CYP2B6, included in the CYP2B isoenzyme family responsible for approximately 25% of the metabolism of marketed drugs [32]. Alongside CYP3A4 and CYP2B6, CAR also modulates a variety of other DMEs and transporters, such as CYP1As, CYP2Cs, UGT1A1, Organic anion transporting polypeptide (OATPs), and MDR1, as well as the aryl hydrocarbon receptor (AhR), another transcription factor [30]. Therefore, CAR is another NR worth screening for when identifying metabolism regulators.

The third transcription factor playing a critical role in drug metabolism is AhR; this receptor belongs to the basic helix-loop-helix family and also functions to regulate enzymes through the addition of xenobiotic ligands [33]. AhR was originally discovered due to its modulating response from exogenous chemicals, such as the contaminant in the Agent Orange herbicide: 2,3,7,8-tetrachlorodibenzo-*p*-dioxin (TCDD) [34]. The main enzymes found to be regulated by AhR are CYP1A1, CYP1A2, Nicotinamide adenine dinucleotide phosphate (NAD(P)H) quinone oxidoreductase (NQO1), aldehyde dehydrogenase 3 (Aldh3a1), UGT1A6, and Glutathione S-Transferase Alpha 1 (GSTA1) [35]. The coverage that PXR, CAR, and AhR achieve is immense and therefore, even studying the three of these alone will gather a lucrative amount of information about a compound’s metabolism.

## 2. In Vitro Metabolism Methods

Several in vitro metabolic techniques have been generated, to activate or simulate metabolism, and used in a compound testing system (Table 1). Based on the purpose of the assay and technique used in the screening, there are many options from which to choose that can add metabolic capability. Each method has its own advantages and disadvantages, which makes selecting the right one for a specific assay important. For example, recombinant metabolic enzymes allow us to study the activity of a single metabolic enzyme. However, our bodies do not contain just one enzyme, so this does not represent the whole picture.

### 2.1. Liver S9 Fractions

Liver S9 fractions, containing both microsomal and cytosolic fractions, are one of the in vitro metabolic activation methods which can be used to test compound activity [58]. S9 fractions include similar metabolic enzymes as hepatocytes, including CYPs, UGTs, aldehyde oxidases, xanthine oxidases, sulfotransferases, methyltransferases, *N*-acetyl transferases, and glutathione transferases [58]. Therefore, S9 fractions can offer a more complete metabolic profile compared with using microsomes or cytosolic fractions alone. Being commercially available, liver S9 fractions are easily accessible and reliable. One of the advantages of using the S9 mix is that co-factors are already included and therefore is ready to use upon purchase [59]. Although this is a tremendous benefit when studying drug metabolism and DDIs in vitro, the S9 mix shows direct toxicity to the cells at the necessary working concentration [60]. This technique then requires additional washing steps to avoid cell damage and death. The requirement of extra washing steps makes it challenging to apply these S9 mixtures into a high-throughput format because most protocols for HTS prefer to use a homogenous assay format to avoid well-to-well variation.

### 2.2. Liver Microsomal Fractions

When it comes to metabolic capability, liver microsomes are often used in an in vitro model [4]. They contain phase I enzymes, such as CYPs, flavin-containing monooxygenases, esterases (i.e., hCE1 and hCE2), amidases, epoxide hydrolases, and phase II enzymes, such as UGTs. Encompassing all of these enzymes is important due to the fact that CYPs and UGTs are responsible for most of the metabolism of marketed drugs [8,19]. One of the advantages of using this system is the long shelf-life that a microsomal fraction has since it can be stored for up to ten years at −70 °C [37]. Since microsomes are a subcellular fraction, the tested compounds do not need to be incorporated into cells or go through the cellular membrane to be metabolized. In addition, microsomes show less cytotoxicity than the S9 mix which makes it easy to apply into an HTS system. However, there are some disadvantages to using microsomes as well; the most significant being that they are not as physiologically relevant as hepatocytes or in vivo models. Another drawback is that they require supplementation with co-factors to activate their metabolism [37].

### 2.3. Liver Cytosolic Fractions

Liver cytosolic fractions include soluble phase I enzymes, such as esterases, amidases, and epoxide hydrolases, as well as soluble phase II enzymes, such as most of the sulfotransferases, glutathione S-transferases, and *N*-acetyltransferases [38]. However, it is important to note that cytosolic fractions do not contain major DMEs such as CYPs. Although CYPs are the most critical enzymes in metabolism, not all metabolism depends on them. Therefore, cytosolic fractions allow us to address the metabolic process for those drugs which are metabolized by soluble enzymes alone. Due to the lack of major metabolizing enzymes, cytosolic fractions are useful to focus on the compounds which require CYP-independent metabolism.

### 2.4. Hepatocytes

Primary hepatocytes represent the most metabolically relevant system which can be used in an assay due to the fact that they are isolated directly from the liver [51]. Since culturing methods and storage affect enzymatic activity [52], the conditions have been optimized to overcome the dedifferentiation process which would lead to a decrease in enzymatic activity [53]. Although the step-by-step isolation process remains the same, there can be a large variation in data between livers from different donors. Each donor can have a large number of single nucleotide polymorphisms in their CYPs [54], and expression levels are also related to the hosts’ life style (e.g., smoking) [61]. However, they still have physiological metabolic activity representative of what happens in vivo and are therefore one of the best techniques in getting accurate metabolic data.

The collagen sandwich model has been an essential in vitro model when studying hepatic drug disposition [62]. In this method, cells are plated on collagen-coated plates with Matrigel added on top to produce a “sandwich” culture [63]. Hepatocytes are in direct contact with a solid extracellular matrix scaffold, such as collagen; hence, this model is representative of the cellular integrity which occurs in the liver [63]. Thus, the model is reliable for studying drug biotransformation, induction, and transporter-mediated biliary excretion.

Cryopreserved hepatocytes [64] are also viable sources of this metabolically competent model and are commercially available. Cryopreserved hepatocytes can lead to successful predictions of in vivo hepatic clearance, indicating an excellent correlation to newly prepared fresh cell cultures [65]. Moreover, the interindividual variability seen with fresh human primary hepatocytes can be irradicated in cryopreserved cells if the same lot of hepatocytes are used for every experiment.

### 2.5. Hepatoma Cell Lines and Terminally Differentiated HepaRG Cells

There are several hepatoma cell lines which have been established, including HepG2 [39,40], HepaRG [41], HLE [40], Transformed Human Liver Epithelial-2 (THLE-2) [42], and Fa2N4 [39]; all of these cell lines are used widely for studying liver toxicity. HepG2 cells, derived from a hepatocellular carcinoma of a 15-year-old Caucasian male [66], have been the most frequently used and are the best-characterized human hepatoma cells due to their ease of use and initial discovery. However, this readily available cell line displays different patterns of enzyme expressions from human primary hepatocytes according to the source and culture conditions used [43,44]. For example, the expression level of CYP3A4, CYP2D6, and CYP2E1 in human primary hepatocytes is 100, 60, and 50 times higher than that in HepG2 cells, respectively [67]. HepaRG cells, another frequently used although relatively new cell line, are terminally differentiated hepatic cells derived from a human hepatic progenitor cell line [48,49]. These cells can differentiate into hepatocyte- or cholangiocyte-like cells depending on culture conditions [68]. In contrast to the HepG2 cell line, HepaRG cells have shown high degrees of similarity with fresh hepatocytes in terms of morphology, mRNA expression profiles for metabolizing enzymes (phases I and II) and transporters [50], and transcription factor activity (AhR, PXR, and CAR) [47,50]. HepaRG cells represent a reliable alternative to human hepatocytes and have become popular liver cells for drug metabolism and toxicity studies. HLE is a hepatocellular carcinoma cell line derived from a 68-year-old male patient [40]. THLE-2 cells, derived from human adult hepatocytes, and Fa2N4 cells, derived from a 12-year-old female donor, were both immortalized by the introduction of the simian virus 40 large T antigen [69,70]. HLE, THLE-2, and Fa2N4 cells are used for metabolic studies, but not as commonly as HepG2 cells.

In contrast with fresh or cryopreserved hepatocytes, these hepatoma cell lines can be transfected with vectors which can mediate DNA or RNA constructs to either express, activate, or knock out a specific gene [71]. This gene-editing characteristic can help to enhance the activity of DMEs, incorporate reporter genes into the cell, and ultimately apply metabolism to previously metabolism-lacking cell lines which can then be used for HTS platforms [5].

### 2.6. Recombinant Enzymes of Phase I and II Drug Metabolism

Several recombinant expression systems have been developed by using Sf9 [72], *Escherichia coli* [73], yeast [74], and mammalian cell models, including the HEK293 cell line [75] which was derived by the transformation of primary cultures of human embryonic kidney cells [76]. The significant advantage of recombinant enzymes is that the activity of one human CYP or UGT can be studied separately to determine a drug’s impact on that specific enzyme without an interference. However, in vivo models do not have isolated systems which have no interaction with other enzymes or factors; they are in contact with many systems at once, and therefore, recombinant enzymes may not mimic physiological conditions. Therefore, recombinant enzymes are not suitable to study the activity of metabolites if the intention is to determine the impact on a physiologically cellular system.

### 2.7. Liver Slices

Utilizing a liver slice is another useful tool when studying metabolism in vitro [77,78]. Unlike other in vitro metabolic systems, it has a physiological condition relevant to the host’s liver. Liver slices contain the complete metabolic machinery with all phase I and phase II DMEs included. Hence, compounds can be examined for all possible metabolic reactions, as in the human body. Containing all the cell subtypes that form the organ, liver slices represent a more relevant in vivo physiological condition than most other approaches. For example, communication between hepatocyte and hepatic progenitor cells will occur, just as in a full-system animal model [55]. One disadvantage of using liver slices is no commercial availability; therefore, they need to be prepared freshly in the research lab [56,57]. Although liver slices are a relevant model, it is difficult to apply them into a high-throughput format due to the difficulty in preparing them.

### 2.8. Monolayer 2D vs. Spheroid 3D Models

Another method commonly used to study drug metabolism is 3D spheroid cell culture [79]. Since cells in our body perform bioactivities under a highly complex 3D microenvironment, this method represents more in vivo circumstances. When using the 3D culture method, cells are grown into spheroids to create a more physiologically relevant system. While spheroids contain multiple layers, 2D cultured cells only form a monolayer. The metabolic activity, including drug metabolism, is also richer in 3D spheroid models than in the 2D culture method [80,81]. The metabolic activity of CYP enzymes including CYP1A2, CYP2B6, and CYP3A4 is higher in 3D spheroid models when compared with 2D cells [45]. Assays with 3D culturing can be used for cell number monitoring, viability, morphology, proliferation, differentiation, migration and invasion of tumor cells into surrounding tissues, angiogenesis formation, immune system modulation, drug metabolism, gene expression, and protein synthesis, as well as many other endpoints [79]. Although the 3D culturing technique has many advantages, the cost is higher and the results are less reproducible than with 2D culturing [46]. Since utilizing 3D cultures are getting more focus and becoming more automated [82], it is possible that the problems with cost and reproducibility will be improved so that HTS options may rise in the future.

## 3. Applying In Vitro Metabolism Methods to Quantitative High-Throughput Screening

### 3.1. Genotoxicity

Genotoxicity occurs when toxicity to genomic DNA happens, potentially leading to genetic mutations [83]. Chemicals classified as “genotoxic” can induce DNA damage [84], which is then repaired by several DNA repair enzymes [85,86,87]. However, cells may fail to repair the damage during the DNA repair process, which increases the risk of tumor formation [88]. Industries have applied several in vitro assays, such as the Ames test [89], the micronucleus test [90], and the comet assay [91] to assess chemically induced DNA damage. Some compounds generate genotoxicity without metabolic activation, whereas other compounds need metabolic activation to produce their genotoxic effect, such as *N*-nitrosodimethylamine [92]. When a compound undergoes metabolic activation, it changes the structure or electric charge of the original chemical [93,94]; given this fact, metabolites are usually more hydrophilic than the parental compounds. These metabolites are more likely to bind to DNA, leading to corrupt replication. In vitro metabolic activation methods are necessary in HTS to determine the effects these metabolites will have on genotoxicity. High-content screening (HCS) provides an insight into the mechanism of action of a genotoxic compound because of its ability to measure multiple parameters at once (e.g., the number of phosho-H2AX (H2A histone family member X) foci and micronucleus) [95,96,97]. When performing micronucleus testing, the assay is performed by co-treating with S9 mix, which will then help to determine if the metabolite is genotoxic. For example, cyclophosphamide shows a higher percentage of micronucleus positive cells when the cells are treated with the S9 mix as opposed to without treatment [84].

### 3.2. Neurotoxicity and Developmental Neurotoxicity

Compounds which have been classified as neurotoxic agents damage the brain or peripheral nervous system. They can induce neurological diseases, including attention-deficit hyperactivity disorder (ADHD), autism, Alzheimer’s disease, and Parkinson’s disease [98,99,100]. A reliable method to test compounds for neurotoxic characteristics is to perform HCS checking cell morphology, or neurite outgrowth [4,101,102]. Neurotoxicity often presents itself by inhibiting neurite outgrowth. Neurite outgrowth is a fundamental process in the differentiation of neurons. It begins at the cell body and extends outward to form functional synapses [103]. One assay, in which neurotoxicity can be assessed, is an acetylcholinesterase (AChE) inhibition assay which can be used in a quantitative HTS (qHTS) platform while inducing metabolic activation by the addition of microsomes [4]. AChE is found in many types of tissues, such as nerve, muscle, and peripheral tissues [104,105]. AChE is responsible for the termination of impulse transmission by rapidly hydrolyzing the neurotransmitter acetylcholine to acetate and choline [106]. After this transformation, the pre-synaptic nerve incorporates choline and combines it with acetyl-CoA to produce acetylcholine through the action of choline acetyltransferase [106]. The inhibition of AChE can induce acetylcholine accumulation in the synaptic space which stimulates nicotinic and muscarinic receptors [106], leading to cholinergic crisis, muscular weakness, fatigue, diarrhea, or salivation [107]. Therefore, AChE inhibitors play an essential role in both toxicology and pharmacology. It is important to replicate what is happening in the body, and therefore when screening, metabolic activation is necessary to identify true AChE inhibitors. For example, chlorpyrifos showed no AChE inhibitory action without microsome treatment, but inhibited AChE when microsomes were co-treated, identifying this compound’s metabolite as a genuine AChE inhibitor [4].

### 3.3. Hepatotoxicity

The liver plays an essential role in drug metabolism; therefore, hepatotoxicity, or liver damage, is a major concern in drug development [108]. Many in vitro assays have been applied to assess the hepatotoxicity of a large set of marketed drugs [109]. These assays employ several cell models, including the HepG2 cell line, terminally differentiated HepaRG cells, and primary hepatocytes [64,110]. Cell viability assays and HCS in HepG2 cells have been developed for hepatotoxicity assessment [111]. However, as stated previously, HepG2 cells are poor detectors of hepatotoxicity induced by reactive metabolites due to their minimal amount of CYP enzymes [112]. Although overexpression of a CYP enzyme by adenoviral transfection can compensate for this marginal expression [5], the HepG2 cell line still lacks phase II enzymatic activity [113]. To overcome this problem, HepaRG cells have become a more readily used source due to their increased metabolic activity [50]. In fact, HepaRG cells show a relatively similar metabolic activity and expression profile as that of human hepatocytes [114,115]. High-content screening of both HepG2 and HepaRG cells can be a promising approach due to the differences in each cell line [116]; by utilizing both cell lines, more potentially toxic compounds can be identified. Recently, liver spheroids have become a popular cellular model to assess compound hepatotoxicity because of their ability to generate a profile similar to that of primary hepatocytes and hepatotoxic profiles for testing compounds [117].

## 4. Future Perspective

The U.S. Tox21 program, which involves the National Institutes of Health (NIH), Environmental Protection Agency (EPA), and Food and Drug Administration (FDA), was established to evaluate thousands of environmental chemicals quickly and efficiently [118]. This program has utilized a qHTS approach to assess a comprehensive 10,000 compound collection of chemicals by a series of biologically and toxicologically relevant in vitro assays [119,120]. Since 2008, more than 70 qHTS methods have been optimized [121]. Although these assays can detect many different endpoints of toxicity, such as genotoxicity [122,123], mitochondrial toxicity [124,125], and stress-related pathways [126,127], most of them are not capable of detecting compounds via metabolic activation. Since most compounds, including drugs, are metabolized in our bodies, in vitro metabolism methods are essential to assess the effect of compounds on our bodies.

Several in vitro metabolism methods have been developed and applied into HTS platforms to compensate for the lack of metabolic activity in cell-based assays. However, those assays cannot completely represent the physiological condition due to the lack of metabolic enzymes or a microenvironment. To represent a more relevant microenvironment, several in vitro metabolizing methods have newly been developed. A method using 3D cell culturing (i.e., spheroids) is one possibility that we can use to assess metabolite activity. This assay has more metabolic capability than a traditional 2D culture model [45]; cells can differentiate in this model, providing cell-to-cell interactions which occur under traditional physiological conditions [81]. Another method that well-represents physiological metabolism is the recently developed organ-on-a-chip (OOAC) technique. OOAC refers to a physiological organ biomimetic system built on a microfluidic chip [128,129]. OOAC combines cell biology, bioengineering, and biomaterial technology allowing us to mimic a specific organ. This technique will allow for greater prediction of the effects of metabolites to each individual organ [130]. Although OOAC can be utilized in an HTS format, there are some challenges to consider, such as automation in dispensing, washing, and monitoring [131]. Another new in vitro metabolism method is the co-culturing technique. The advantage of this method is that it can mimic physiological conditions due to the capability of culturing different types of cells in one well [132]. For example, to evaluate compounds’ developmental toxicity, mesenchymal stem cells and endothelial cells can be co-cultured in an angiogenesis assay [133,134]. Organoids, or 3D culture primary cells, are another future possibility in HTS. While previously mentioned 3D spheroids are derived from cancer cells, organoids are derived from adult organs or pluripotent stem cells [135]. As the differentiation of stem cells occurs, the organoids become more complex. Considering that gastrointestinal organoids have already been applied in an HTS format [136], it is possible that liver organoids can also be designed in the same way, giving us another trustworthy in vitro metabolism method in the future.

HTS methods are a useful tool to evaluate the activity of compounds quickly and efficiently. However, due to the conflict between simplifying HTS assays and the complexity of our bodies, assays in an HTS format are frequently missing relevant physiological components. The main issue is the lack of metabolic capability in the current formats. In other words, only the parent compound of a chemical was previously assessed in most HTS assays. To overcome this problem, applying the S9 mix, microsomes, or hepatocytes into screening methods is a promising start. New in vitro metabolic technologies, which well-represent physiological conditions, should be used in future screening to fully encompass the activity of drugs and chemical compounds. Knowing the effects of a drug or chemical compound helps to determine and better understand the mechanism of action behind each compound’s specific toxicity.

## Figures and Tables

**Table 1:** Comparison of metabolic components from protein to cells.

Metabolic Components/Cells [References]	Pros	Cons
Recombinant protein [36]	Single metabolizing protein focus HTS compatible	Missing cellular component
Liver microsomes [37,38]	Less cytotoxicity Major phase I enzymes such as CYPs Commercially available Short protocol HTS compatible	Less phase II enzymes such as SULT
Liver cytosol [38]	Major phase II enzymes such as GST Commercially available	No CYPs
Liver S9 fractions [38]	Physiological phase I and II enzymes Commercially available Short protocol	High cytotoxicity
Hepatoma cell lines (HepG2, HLE, THLE-2, and Fa2N4) [39,40,41,42,43,44]	Commercially available Well-developed protocols HTS compatible	Less metabolic activity
3D cell culture [45,46]	Higher metabolic activity	Time consuming Expensive
Terminally differentiated HepaRG cells [47,48,49,50]	Commercially available Higher metabolic activity	Expensive
Hepatocytes, liver slices [51,52,53,54,55,56,57]	Commercially available Well-developed protocol All metabolizing enzymes	Lot-to-lot variation Time consuming Expensive

## Abbreviations

AChE	Acetylcholinesterase
AhR	Aryl hydrocarbon receptor
CAR	Constitutive androstane receptor
CYP	Cytochrome p450
DDI	Drug–drug interaction
DME	Drug-metabolizing enzyme
NR	Nuclear receptor
OOTC	Organ-on-a-chip
PXR	Pregnane X receptor
qHTS	Quantitative high-throughput screening
UGT	Glucuronosyltransferase
2D	Two dimensional
3D	Three dimensional
